# The biocompatibility and antifungal effect of *Rosmarinus officinalis* against *Candida albicans* in *Galleria mellonella* model

**DOI:** 10.1038/s41598-022-19425-9

**Published:** 2022-09-16

**Authors:** Vanessa Marques Meccatti, Lívia Mara Alves Figueiredo-Godoi, Thaís Cristine Pereira, Patrícia Michelle Nagai de Lima, Amjad Abu Hasna, Lavinia Barbosa Senna, Maria Cristina Marcucci, Juliana Campos Junqueira, Luciane Dias de Oliveira

**Affiliations:** 1 Department of Biosciences and Oral Diagnosis, Institute of Science and Technology, São Paulo State University (ICT-UNESP), São José dos Campos, SP Brazil; 2 Department of Restorative Dentistry, Endodontics Division, Institute of Science and Technology, São Paulo State University (ICT-UNESP), Av. Eng. Francisco José Longo Avenue 777, São José dos Campos, SP CEP 12245-000 Brazil

**Keywords:** Microbiology, Medical research

## Abstract

This study was performed to evaluate the biocompatibility and antifungal effect of *Rosmarinus officinalis* against *Candida albicans* in *Galleria mellonella* model. Five different concentrations of *R. officinalis* glycolic extract (50; 25; 12.5 e 6.25 mg/mL) were used to evaluate its biocompatibility in *G. mellonella* model, in which the nystatin suspension (100; 50; 25; 12.5 e 6.25%) was used as a control group. The antifungal action of *R. officinalis* glycolic extract was evaluated on *C. albicans* for 72, 48 and 12 h at two different phases: (1) using the extract as therapeutic agent; and (2) using the extract as prophylactic agent. PBS was used as a negative control group. *G. mellonella* survival curves were plotted using the Kaplan–Meier method and statistical analysis was performed using the log-rank test (Mantel–Cox) and the significance level was set at (α ≤ 0.05). There was no significant difference among the groups in which all were biocompatible except of a significant death rate of 26.6% with nystatin 100%. In phase 1, it was found that after 7 days, there was no statistically significant difference among the prophylactic treatment groups. In phase 2, the groups of *R. officinalis* 6.25 mg/mL for 72 h and *R. officinalis* of 12.5 mg/mL for 24 h promoted the survival rate of the larvae in comparison with the control group with a significant difference (p = 0.017) and (p = 0.032) respectively. Therefore, *R. officinalis* extract is biocompatible in different concentrations and can be used as a prophylactic agent against fungal infection.

## Introduction

Oral candidiasis is a fungal infection of the oral cavity^1^, manifested as superficial mucosal lesions, or deep and highly severe lesions due to immune suppression^2^. It is caused by *Candida* species, principally *Candida albicans*^3^, which has resistance to a range of antifungal drugs^4^ because of their uncontrolled use^5^. Besides, *C. albicans* was found in the root canal system of teeth with periapical lesions^6^ and has a potential role in the development of the periodontal diseases^7,8^.

Phytotherapy was indicated for the treatment of some oral conditions because of its biocompatibility, antimicrobial, anti-inflammatory and antioxidant properties^9–12^. *Rosmarinus officinalis* L. popularly called rosemary, is an ancient shrub belonging to the family *Lamiaceae* and originated from the Mediterranean region^13^. This herbal medicine has promising biological effects including antimicrobial, antibiofilm, anti-inflammatory, antioxidant, anti-ulcer, antiviral and anticancer effects^14–18^, besides, it improves memory performance and sleep quality, and reduces anxiety and depression^19^.

The antifungal effect of *R. officinalis* was tested against *C. albicans, C. dubliniensis, C. glabrata, C. krusei,* and *C. tropicalis* where it was as effective as nystatin^20^. However, to the best of our knowledge, there is no studies that evaluated the antifungal effect against *C. albicans* in *Galleria mellonella* model like other herbal medicines^21,22^ as in vivo models provides an opportunity to assess the prophylactic potential of a compound acting on the host’s immune system^23^. Besides, *G. mellonella* is an adequate an invertebrate model organism to test the antimicrobial action over diverse materials over microorganisms like streptococcus, *Escherichia coli*, and *Klebsiella pneumoniae*^24–26^.

This study was elaborated to understand the antifungal effect of *R. officinalis* and is trying to present this herbal medicine as an alternative medication for oral candidiasis, intracanal medication, endodontic irrigant, or toothpaste and mouthwash component. Therefore, the aim of this study was to evaluate the antifungal effect of *R. officinalis* against *C. albicans* and its biocompatibility in *G. mellonella* model. The null hypothesis was that the *R. officinalis* has no antifungal effect and it is not biocompatible.

## Materials and methods

### Plant extract and bacterial strain

*Rosmarinus officinalis* glycolic extract was obtained of the flowers and leaves of rosemary and formulated at a concentration of 20% (200 mg/mL of the extract) in propylene glycol (Mapric^®^, São Paulo, SP, Brazil). The extracts were not manipulated in our laboratory, instead they were obtained in a commercial form composed of essential oil made up of terpenic derivatives (pinene, camphene, free borneol, acetate, cineole, camphor); sesquiterpenes; oleanolic acid; tannin; bitter substances; acid saponin and glucoside compounds according to the manufacturer (Mapric^®^, São Paulo, SP, Brazil).

A standard strain of *C. albicans* (ATCC 18804) was used in this study. The microorganisms were frozen in BHI broth containing 20% glycerol at − 80 °C. At the moment of use, the microorganisms were seeded on CHROM agar to guarantee the absence of contamination. Only *C. albicans* colonies that showed green color were used in this study, in which were cultured on Sabouraud Dextrose agar (Himedia Laboratories, Munbai, India) using the depletion technique and incubated for 48 h at 37 °C. Then, the colonies were transferred to the Yeast extract–Peptone–Dextrose (YPD) broth medium and incubated at 37 °C for 24 h.

### Inoculum preparation and *G. mellonella* selection

The inoculum was prepared and standardized following an anterior protocol reported in the literature^27^. After 24 h incubation in YPD broth, the fungal cells were isolated by centrifugation at 5000 rpm for 10 min, and the supernatant was discarded. The remaining deposit was resuspended in PBS, and vortexed for 30 s. This process was repeated two more times. The standardization of the inoculum was performed using a hemocytometer at 10^6^ cells/larva to be used for the following tests.

*Galleria mellonella* larvae were obtained from the Invertebrate Laboratory belonging to the Microbiology and Immunology Department of the Institute of Science and Technology of São Paulo State University (ICT-Unesp). Only larvae (I) weighing from 250 to 300 mg; (II) in the last larval stage; (III) light colored; and (IV) without spots or dark pigments in their cuticle were used in this study to avoid the involvement of a probable infectious process.

The larvae cleaning and feeding were carried out three times a week. The diet was based on wax, and the ration was composed of 25% cornmeal, 15% brewer’s yeast, 10% soy flour, 10% skimmed milk, 20% honey and 20% glycerol, prepared in-house. The larvae were kept at 25 °C until use. After receiving the treatment, the larvae were not fed.

### Evaluation of toxicity in *G. mellonella* model

Four different concentrations (50; 25; 12.5 and 6.25 mg/mL) of *R. officinalis* glycolic extract at 20% diluted in Phosphate Buffered Saline (PBS) and five different concentrations (100; 50; 25; 12.5 and 6.25%) of nystatin (oral suspension 100,000 IU/mL—Germed) diluted in sterile distilled water were evaluated. The extracts were inoculated in the last right proleg of each larva totaling the n = 15 for each concentration. PBS and another group with no injection were used as control groups.

An aliquot of 10 µL of each concentration was inoculated using Hamilton syringes (Hamilton Inc., USA), then the larvae were kept in Petri dishes at 37 °C in the dark, without nutrition. After 24 h of inoculations, the number of dead *G. mellonella* larvae was recorded daily until 168 h (7 days) for analysis of the survival curve. Larvae were considered dead when they presented no movement after touch. After obtaining non-toxic concentrations of the plant extract and nystatin in *G. mellonella*, these concentrations were used to evaluate the antifungal action of *R. officinalis* L. on *C. albicans.*

### Evaluation of antifungal effect of *R. officinalis* L. on* C. albicans*

After determining the non-toxic concentrations of the plant extract, the larvae were randomly divided into six groups (n = 15) and treated according to the following protocols at phase I of the test:

- Group 1 (Ro 25 mg/mL for 24 h + *C. albicans*): the larvae were inoculated with 10 µL of *R. officinalis* extract (25 mg/mL) in the last right proleg, and after 24 h were inoculated with 10 µL *C. albicans* inoculum in the last left proleg.
- Group 2 (PBS for 24 h + *C. albicans*): the larvae were inoculated with 10 µL of PBS in the last right proleg, and after 24 h were inoculated with 10 µL *C. albicans* inoculum in the last left proleg.
- Group 3 (*C. albicans* for 1 h + Ro 25 mg/mL): the larvae were inoculated with 10 µL *C. albicans* inoculum in the last right proleg, and after 1 h were inoculated with 10 µL of *R. officinalis* extract (25 mg/mL) in the last left proleg.
- Group 4 (*C. albicans* for 1 h + PBS): the larvae were inoculated with 10 µL *C. albicans* inoculum in the last right proleg, and after 1hwere inoculated with 10 µL of PBS in the last left proleg.
- Group 5 (*C. albicans* for 1 h + 25% nystatin): the larvae were inoculated with 10 µL *C. albicans* inoculum in the last right proleg, and after 1hwere inoculated with 10 µL of 25% nystatin in the last left proleg.
- Group 6 (PBS for 1 h + PBS): the larvae were inoculated with 10 µL PBS in the last right proleg, and after 1hwere inoculated with 10 µL of PBS in the last left proleg.

Groups (Ro 25 mg/mL for 24 h + *C. albicans*) and (PBS for 24 h + *C. albicans*) were simulating the prophylactic effect, in which the extracts were inoculated before the infection exists. However, in groups (*C. albicans* for 1 h + Ro 25 mg/mL), (*C. albicans* for 1 h + PBS) and 5, the treatment was applied after the infection, simulating therapeutic effect. Lastly, group (PBS for 1 h + PBS) was considered as a negative control group. After the injections, the larvae were kept in Petri dishes at 37 °C in the dark. The number of dead larvae was observed and recorded daily for 7 days to obtain the survival curve.

Posteriorly, phase II was performed in which the groups were divided as the following:

- Group 7A: (Ro 12.5 mg/mL for 72 h + *C. albicans*): the larvae were inoculated with 10 µL of *R. officinalis* extract (12.5 mg/mL) each 24 h totaling 3 doses in different prolegs, after 24 h were inoculated with 10 µL *C. albicans* inoculum in a different proleg.
- Group 7B: (Ro 6.25 mg/mL for 72 h + *C. albicans*): the same of group (Ro 12.5 mg/mL for 72 h + *C. albicans*). However, the extract concentration was of 6.25 mg/mL.
- Group 8A: (Ro 12.5 mg/mL for 48 h + *C. albicans*): the larvae were inoculated with 10 µL of *R. officinalis* extract (12.5 mg/mL) each 24 h totaling 2 doses in different prolegs, after 24 h were inoculated with 10 µL *C. albicans* inoculum in a different proleg.
- Group 8B: (Ro 6.25 mg/mL for 48 h + *C. albicans*): the same of group (Ro 12.5 mg/mL for 48 h + *C. albicans*). However, the extract concentration was of 6.25 mg/mL.
- Group 9A: (Ro 12.5 mg/mL for 24 h + *C. albicans*): the larvae were inoculated with 10 µL of *R. officinalis* extract (12.5 mg/mL) (1 dose), after 24 h were inoculated with 10 µL *C. albicans* inoculum in a different proleg.
- Group 9B: (Ro 6.25 mg/mL for 24 h + *C. albicans*): the same of group (Ro 12.5 mg/mL for 24 h + *C. albicans*). However, the extract concentration was of 6.25 mg/mL.
- Group 10: (PBS for 24 h + *C. albicans*): the larvae were inoculated with 10 µL of PBS, after 24 h were inoculated with 10 µL *C. albicans* inoculum in a different proleg.
- Group 11: (PBS for 24 h + PBS): the larvae were inoculated with 10 µL of PBS, after 24 h were inoculated with 10 µL *PBS* in a different proleg.

Phase 2 was performed to evaluate the difference between 1, 2 and 3 doses of the extract for prophylactic effect, in which the extracts were inoculated before the infection exists as in groups (Ro 12.5 mg/mL for 72 h + *C. albicans*), (Ro 6.25 mg/mL for 72 h + *C. albicans*), (Ro 12.5 mg/mL for 48 h + *C. albicans*), (Ro 6.25 mg/mL for 48 h + *C. albicans*), (Ro 12.5 mg/mL for 24 h + *C. albicans*) and (Ro 6.25 mg/mL for 24 h + *C. albicans*). Groups (PBS for 24 h + *C. albicans*) and (PBS for 24 h + PBS) were considered as control groups. After the injections, the larvae were kept in Petri dishes at 37 °C in the dark. The number of dead larvae was observed and recorded daily for 7 days to obtain the survival curve.

### Statistical analysis

*Galleria mellonella* survival curves were plotted using the Kaplan–Meier method and statistical analysis was performed using the log-rank test (Mantel–Cox). The analyzes were performed using the GraphPad Prism statistical software and the significance level was set at (α < 0.05).

## Results

### Evaluation of toxicity in *G. mellonella* model

**Figure 1:**
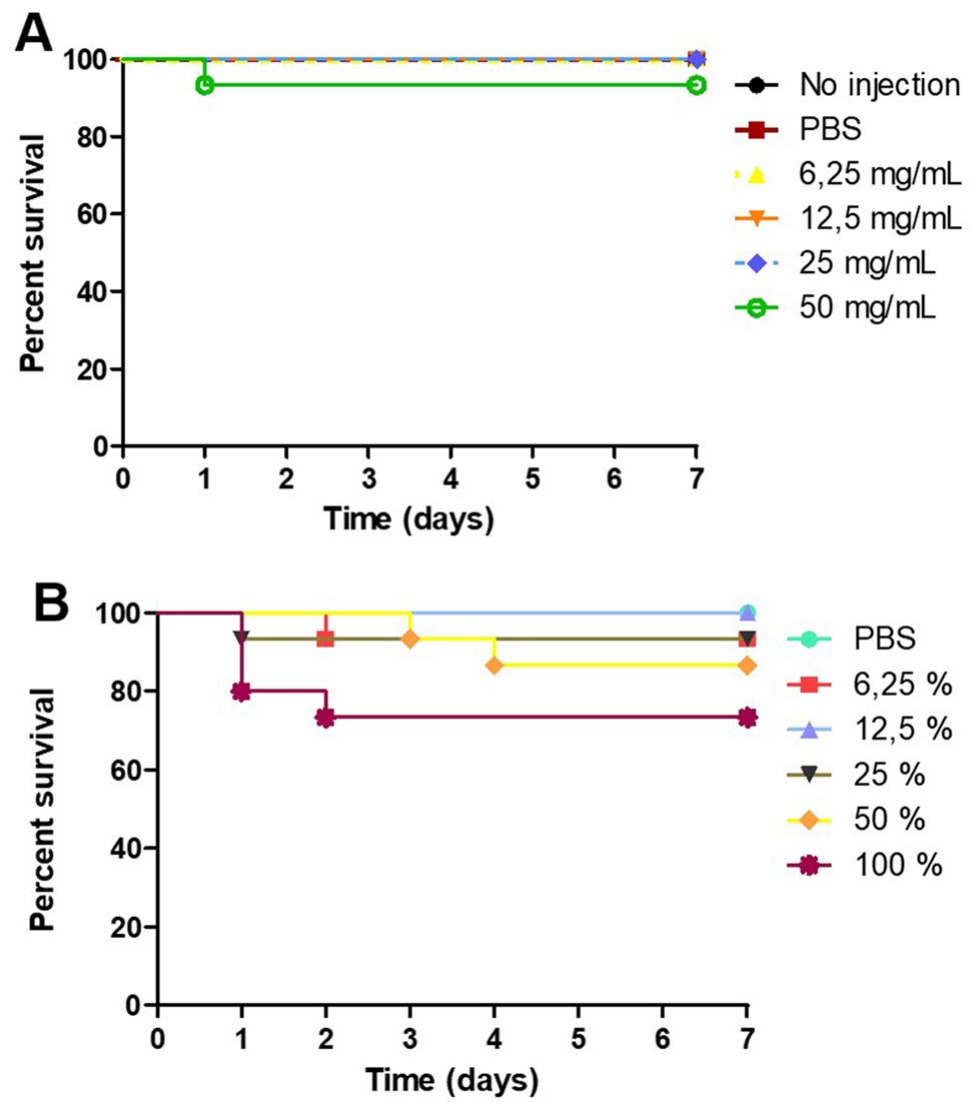
*Galleria mellonella* survival curve after inoculation of *Rosmarinus officinalis* glycolic extract and nystatin in different concentrations. (**A**) To compare the biocompatibility of different concentrations of the *Rosmarinus officinalis* glycolic extract with the control group (PBS). (**B**) To compare the biocompatibility of different concentrations of the nystatin with the control group (PBS).

In the control groups and *R. officinalis* extracts (6.25, 12.5 and 25 mg/mL), all larvae survived after 7 days. *R. officinalis* extracts (50 mg/mL) caused death rate of 6.6% of the larvae. However, there was no significant difference among the groups (Fig. 1A).

Conversely, PBS and nystatin (6.25 and 12.5 mg/mL) where biocompatible, however, there was a death rate 6.6% with nystatin 25%, 13.3% with nystatin 50% and 26.6% with nystatin 100% (Fig. 1B) with p value = 0.034.

### Evaluation of antifungal effect of *R. officinalis* L. on* C. albicans*

**Figure 2:**
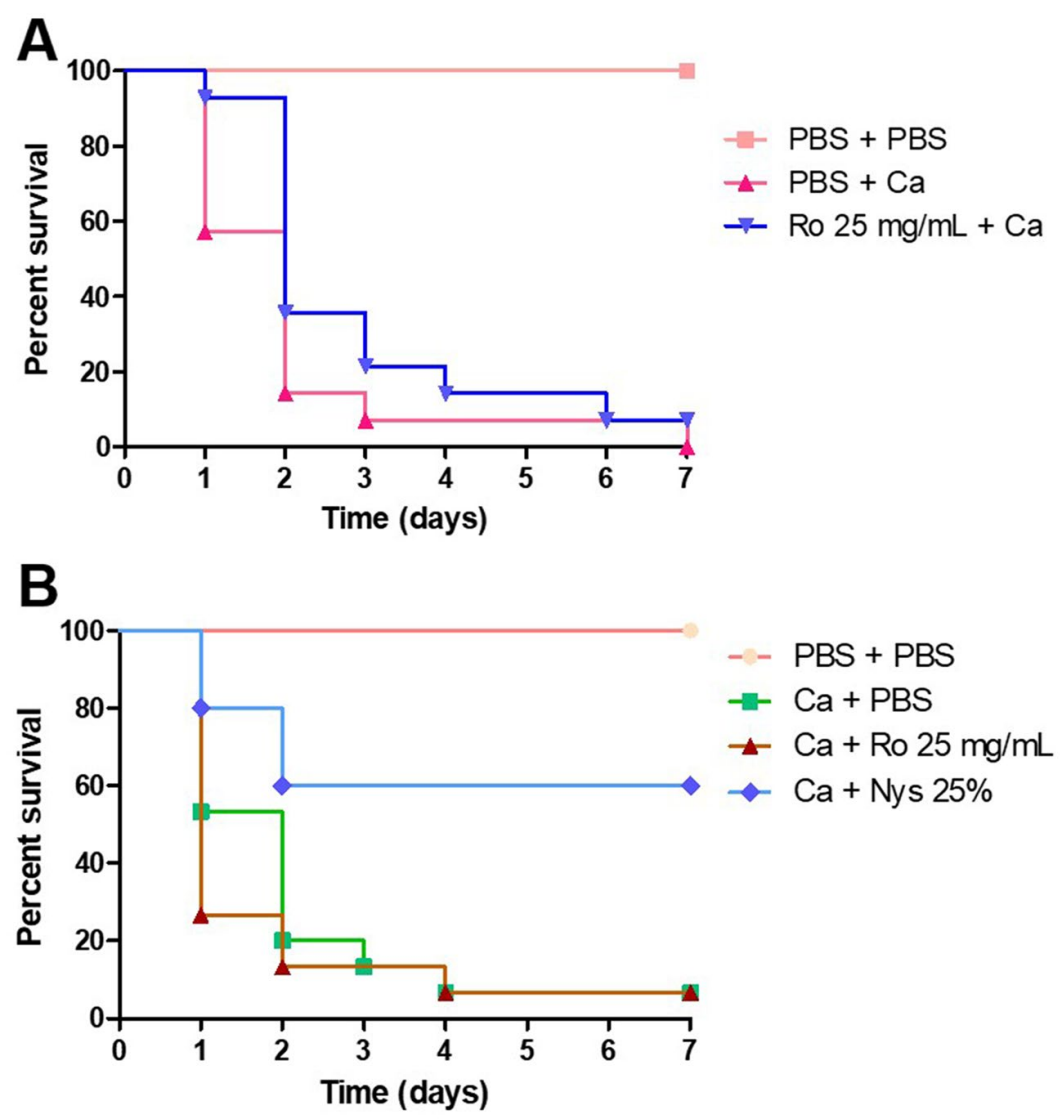
*Galleria mellonella* survival curve after treatment with different groups. (**A**) To compare the antifungal action of the prophylactic groups with the control group (PBS + PBS). (**B**) To compare the antifungal action of the therapeutic groups with the control group (PBS + PBS). *Ca Candida albicans*, *Ro Rosmarinus officinalis*, *Nys* Nystatin.

When evaluating the prophylactic treatment groups, it was found that group (Ro 25 mg/mL for 24 h + *C. albicans*) promoted an increase in the survival of the larvae in comparison with group (PBS for 24 h + *C. albicans*) in the first 24 h in which the survival rate was 57.2% with the group (PBS for 24 h + *C. albicans*); 92.9% with the group (Ro 25 mg/ mL for 24 h + *C. albicans*); and 100% with the control group (PBS for 1 h + PBS). After 7 days, there was no statistically significant difference among the prophylactic treatment groups (Fig. 2A).

When evaluating the therapeutic treatment groups, there was no significant difference between groups (*C. albicans* for 1 h + Ro 25 mg/mL) and (*C. albicans* for 1 h + PBS). However, group (*C. albicans* for 1 h + 25% nystatin) presented a significant difference in comparison with the groups (*C. albicans* for 1 h + Ro 25 mg/mL) and (*C. albicans* for 1 h + PBS) as it promoted the survival rate of the larvae up to 60% after 7 days (Fig. 2B).

**Figure 3:**
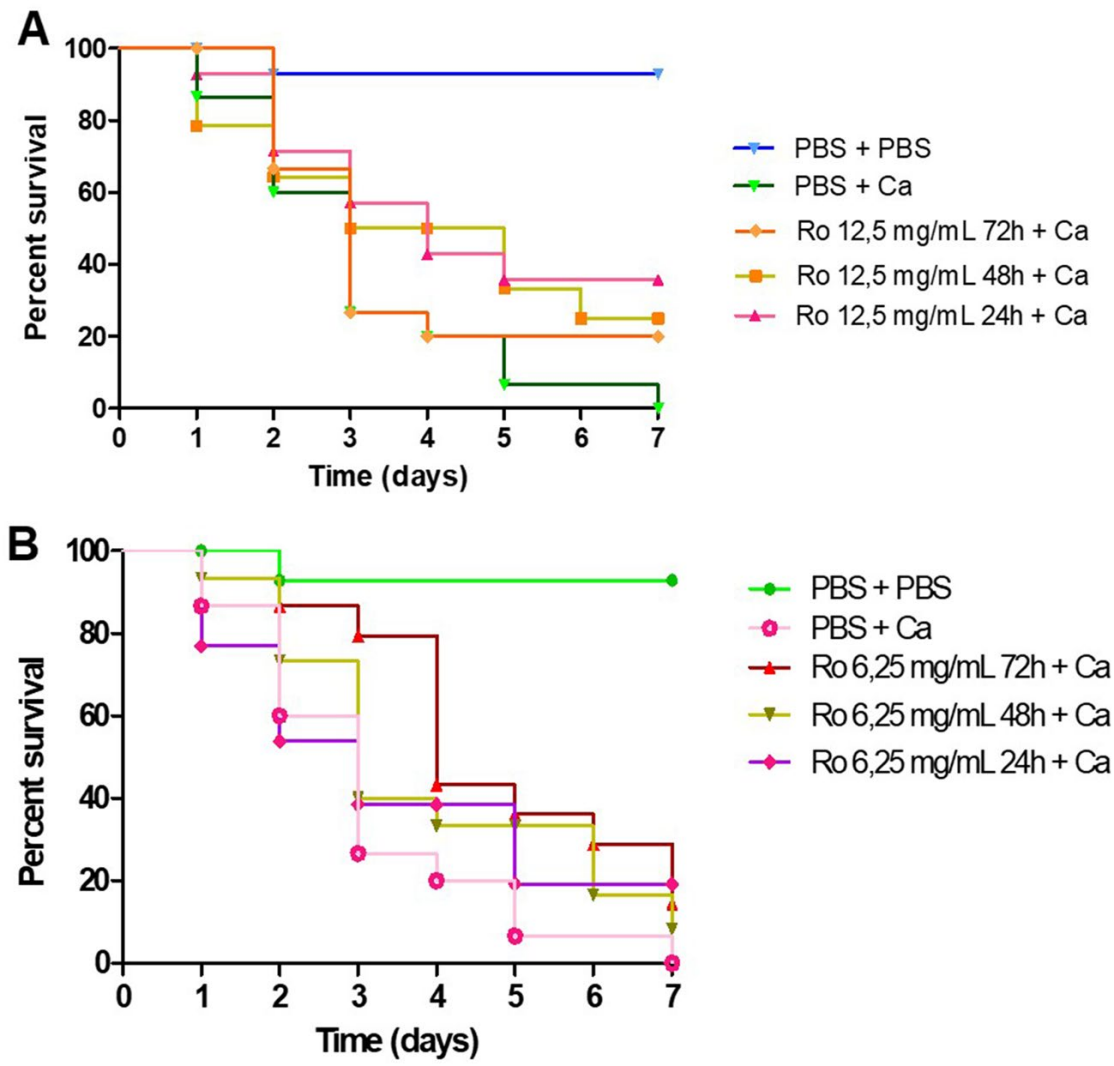
*Galleria mellonella* survival curve after treatment with different groups. (**A**) To compare the antifungal action of the prophylactic groups of 12.5 mg/mL with the control group (PBS + PBS). (**B**) To compare the antifungal action of the prophylactic groups of 6.25 mg/mL with the control group (PBS + PBS). *Ca Candida albicans*, *Ro Rosmarinus officinalis*, *Nys* Nystatin.

In phase 2 of the analysis, there was a significant difference between the group (Ro 12.5 mg/mL for 24 h + *C. albicans*) and (PBS for 24 h + *C. albicans*) (p = 0.032); however, there was no significant difference between the groups (Ro 12.5 mg/mL for 48 h + *C. albicans*) and (PBS for 24 h + *C. albicans*) (p = 0.09). In Fig. 3A, it was observed that in the 4th day, the survival rate with group (PBS for 24 h + *C. albicans*) was 26.7% and with the group (Ro 12.5 mg/mL for 24 h + *C. albicans*) was 57.2%.

Besides, there was a significant difference between the group (Ro 6.25 mg/mL for 72 h + *C. albicans*) and (PBS for 24 h + *C. albicans*) (p = 0.017). After 7 days, 100% of the larvae were dead with the group (PBS for 24 h + *C. albicans*), and the survival rate was 35% with the group (Ro 12.5 mg/mL for 24 h + *C. albicans*). In the 3rd day, it was verified that the survival rate was of 79.4% with the group (Ro 6.25 mg/mL for 72 h + *C. albicans*) and 26.6% with the group (PBS for 24 h + *C. albicans*). After 7 days, all larvae in the group (PBS for 24 h + *C. albicans*) had died and the survival rate in the group (Ro 6.25 mg/mL for 72 h + *C. albicans*) was 14.4% (Fig. 3B).

The groups (Ro 6.25 mg/mL for 72 h + *C. albicans*) and (Ro 12.5 mg/mL for 24 h + *C. albicans*) promoted the survival rate of the larvae in comparison with the group (PBS for 24 h + *C. albicans*) with a significant difference (p = 0.017) e (p = 0.032) respectively.

## Discussion

*Candida albicans* are opportunistic microorganisms, that may cause a fungal infection, as in situations where the immune system is compromised like in coronavirus disease 19 (COVID-19) infections caused by severe acute respiratory syndrome of coronavirus-2 (SARS-CoV2), making the clinical course of these patients more complicated^28,29^. The aim of this study was to evaluate the antifungal effect of *R. officinalis* against *C. albicans* and its biocompatibility in *G. mellonella* model. It was found that the extract has an effective antifungal effect against *C. albicans* and is biocompatible thus the null hypothesis was rejected.

In this study it was found that, *R. officinalis* extracts (6.25, 12.5 and 25 mg/mL) were as biocompatible as the control group in which all the larvae survived after 7 days. However, *R. officinalis* extract of 50 mg/mL caused death rate of 6.6% of the larvae. Still, there was no significant difference among the groups (Fig. 1A). To the best of our knowledge, there are no studies in the literature that evaluated the toxicity of *R. officinalis* in *G. mellonella* model. However, in the literature, it was found that rosemary products such as extracts and essential oils do not present toxicity over fibroblasts and macrophages^30,31^. Besides, some reports on the use of the *R. officinalis* extract in murine models were found: the administration of a single dose of *R. officinalis* extract at a concentration of 2000 mg/kg in rats was described to be safe^32^ and the single lethal dose median (LD50) of rosemary essential oil in rats was 4753.3 mg/kg^33^.

*Galleria mellonella* model is an adequate model to test the toxicity of medicinal plants, in a recent study, the model was used to test seven well known plants from Cameroon using the *G. mellonella* larvae, in which it was found that the plant materials and the extracts type (ethanolic or aqueous) affect the toxicity^34^. In the present study, the dead larvae counting was recorded until 7 days as reported in anterior studies in the literature^27,35^.

The results of the present study are significant since there are no reports in the literature about the biocompatibility of the commercially used nystatin suspension on *G. mellonella*. The *R. officinalis* extract of 50 mg/mL presented a death rate of only 6.6% of the larvae, compared to nystatin, the main antifungal administered in cases of oral candidosis, which presented a death rate of 26.6% when administered alone.

On the other hand, the antifungal action of *R. officinalis* extracts were evaluated in *G. mellonella* model as in the protocol of another study^36^. In this study, the therapeutic inoculation with the antifungal agent was performed after 1 h of infection to maintain a survival rate of 60% of the larvae; however, in another study the antimicrobial treatment was performed 3 h after infection^37^.

In phase 1 of the present study, it was found that there was no significant difference between groups (*C. albicans* for 1 h + Ro 25 mg/mL) and (*C. albicans* for 1 h + PBS), the therapeutic treatment groups. However, group (*C. albicans* for 1 h + 25% nystatin) presented a significant difference in comparison with the groups (*C. albicans* for 1 h + Ro 25 mg/mL) and (*C. albicans* for 1 h + PBS) as it promoted the survival rate of the larvae up to 60% after 7 days (Fig. 2B). In the literature, *R. officinalis* L. extract showed antifungal effect on *Candida* spp. without a significant difference with nystatin in vitro^20^; however, they were not tested in in *G. mellonella* model as in the present study.

In a comparative analysis, it is possible to verify that the prophylactic protocol of group (Ro 25 mg/mL for 24 h + *C. albicans*) promoted greater survival of the larvae, since on the first day of counting, most of the larvae survived and then the survival curve declined. However, even at the end of the 7th day of counting, there were still surviving larvae in the group previously treated with extract. When the prophylactic group was compared to the group (PBS for 24 h + *C. albicans*) with the p value = 0.08.

In another study, the prophylactic potential of using *Lactobacillus* spp. on *G. mellonella* infected by *Candida* spp. was verified 24 h before the infection, while the conventional treatment occurred after 30 min of the yeast inoculation. At the end of the study, the results showed benefits in the use of the prophylactic protocol because the larvae that were inoculated with *Lactobacillus* spp. prophylactically had a longer survival compared to those who received conventional treatment^38^. This finding corroborates our results, in which the group that received prophylactic treatment (24 h before infection) with 25 mg/mL of *R. officinalis* extract had greater survival than the group with conventional treatment.

In phase 2 of this study, the groups (Ro 6.25 mg/mL for 72 h + *C. albicans*) and (Ro 12.5 mg/mL for 24 h + *C. albicans*) of 12.5 mg/mL for 24 h as prophylactic treatments promoted the survival rate of the larvae in comparison with the group (PBS for 24 h + *C. albicans*) with a significant difference (p = 0.017) e (p = 0.032) respectively.

Almeida et al. evaluated the antifungal potential of pomegranate in vivo on *G. mellonella* infected by *C. albicans*. After comparative analysis of the prophylactic and therapeutic protocols using the plant extract, the study reported that prophylaxis with pomegranate had better results^39^, which is in line with our findings. In their study, only one concentration of pomegranate was tested, while in the present study, a total of three concentrations of rosemary were tested. The same study verified that with multiple administrations of the plant extracts before fungal infection the percentage of larvae survival was higher when compared to prophylaxis in a single dose.

The initial mechanisms of the larval immune response to the infectious process can be divided into two phases: (I) phase 1 corresponds to the first 6 h, in which there is a decrease in fungal load, which can be correlated with an increase in hemocyte density and an increase in antimicrobial peptides and immune system proteins; (II) phase 2, from 6 to 24 h, yeast cells proliferate and the number of hemocytes decreases, resulting in larval death^40^. The results of the present study suggest that these deleterious effects on the immune system of the larvae caused by *C. albicans* infection were attenuated by the prophylactic administration of rosemary extract, since the antimicrobial activity of the plant extract is already well founded^41^. The present study elucidated important findings on the application of *R. officinalis* extract against *C. albicans*, mainly on the prophylactic action. The contributions of this in vivo study are relevant and notorious for the advancement of research on the biological properties of this plant.

## Conclusion

The prophylactic antifungal effect found in this study suggest the use of *R. officinalis* extract as an alternative medication for fungal infection.

## Data Availability

The data used to support the findings of this study are available upon request with the corresponding author d.d.s.amjad@gmail.com.
